# Supplementary material for: Secretion of an Argonaute protein by a parasitic nematode and the evolution of its siRNA guides
Source: Nucleic Acids Res. 2019 Mar 1;47(7):3594–606. doi: 10.1093/nar/gkz142 (PMC6468290; doi:10.1093/nar/gkz142)
Supplement: Supplementary Data [file gkz142_supplemental_files.zip › new-Supplemental Table 1.docx]

**Supplemental Table 1:** *Heligmosomoides bakeri* genome assembly

| **Feature** | ***Heligmosomoides bakeri*** **genome assembly v2.0** | ***Heligmosomoides bakeri*** **genome assembly v1.0** |
| --- | --- | --- |
| Reference | This work | WTSI |
| Span (Mb) | 696 | 560 |
| G+C content (%) | 45.6 | 45.0 |
| Scaffold / contig N50 (kb) | 179.6 / 42.6 | 35.8 / 12.8 |
| Number of contigs | 23647 | 44728 |
| Genome CEGMA complete / partial (%) | 88.7 / 8.1 | 78.8 / 18.1 |
| Genome BUSCO (Nematoda) complete / partial (%) | 87.1 / 7.2 | 67.8 / 10.7 |
| Genome BUSCO (Eukaryota) complete / partial (%) | 87.8 / 1.7 | 74.3 / 8.9 |
| Transcriptome mapping | 96.3% | 72.3% |
| Number of protein-coding genes | 24371 | 27459 |
