# Supplementary material for: Secretion of an Argonaute protein by a parasitic nematode and the evolution of its siRNA guides
Source: Nucleic Acids Res. 2019 Mar 1;47(7):3594–606. doi: 10.1093/nar/gkz142 (PMC6468290; doi:10.1093/nar/gkz142)
Supplement: Supplementary Data [file gkz142_supplemental_files.zip › new-SupplementaryTable2.docx]

| **Prefix** | **Species** | **Source** |  |
| --- | --- | --- | --- |
| ACEYL | *Ancylostoma ceylanicum* | WBPS8 PRJNA231479 |  |
| ACANT | *Angiostrongylus cantonensis* | WBPS8 PRJEB493 |  |
| ACOST | *Angiostrongylus costaricensis* | WBPS8 PRJEB494 |  |
| CAFRA | *Caenorhabditis afra* | CGP2 JU1286 |  |
| CBRIG | *Caenorhabditis briggsae* | WBPS8 PRJNA10731 |  |
| CCAST | *Caenorhabditis castelli* | CGP2 JU1956 |  |
| CELEG | *Caenorhabditis elegans* | WBPS8 PRJNA13758 |  |
| CSP1 | *Caenorhabditis monodelphis* | CGP2 JU1667 |  |
| CSP38 | *Caenorhabditis* sp. 38 | CGP2 JU2809 |  |
| CTROP | *Caenorhabditis tropicalis* | WBPS8 PRJNA53597 |  |
| DVIVI | *Dictyocaulus viviparus* | WBPS8 PRJEB5116 |  |
| HCONT | *Haemonchus contortus* | WBPS8 PRJEB506 |  |
| HPLAC | *haemonchus placei* | WBPS8 PRJEB509 |  |
| HPOLY | *Heligmosomoides polygyrus* | EDI v2 |  |
| HBACT | *Heterorhabditis bacteriophora* | WBPS8 PRJNA13977 |  |
| NAMER | *Necator americanus* | WBPS8 PRJNA72135 |  |
| NBRAS | *Nippostrongylus brasiliensis* | WBPS8 PRJEB511 |  |
| ODENT | *Oesophagostomum dentatum* | WBPS8 PRJNA72579 |  |
| OTIPU | *Oscheius tipulae* | EDI v2 |  |
| PPACI | *Pristionchus pacificus* | WBPS8 PRJNA12644 |  |
|  |  |  |  |

**Supplementary Table 2**: Proteomes included in the OrthoFinder clustering analysis. Prefix: prefix used for protein sequences. Species: species name. Source: source of protein files. WBPS8: WormBase ParaSite 8. EDI. CGP2: Caenorhabditis Genome Project *v2.*
